# Genome-Wide Analysis of the C2 Domain Family in Soybean and Identification of a Putative Abiotic Stress Response Gene *GmC2-148*

**DOI:** 10.3389/fpls.2021.620544

**Published:** 2021-02-16

**Authors:** Yue Sun, Juan-Ying Zhao, Yi-Tong Li, Pei-Gen Zhang, Shu-Ping Wang, Jun Guo, Jun Chen, Yong-Bin Zhou, Ming Chen, You-Zhi Ma, Zheng-Wu Fang, Zhao-Shi Xu

**Affiliations:** ^1^College of Agriculture, Yangtze University, Hubei Collaborative Innovation Center for Grain Industry, Engineering Research Center of Ecology and Agricultural Use of Wetland, Ministry of Education, Jingzhou, China; ^2^Institute of Crop Science, Chinese Academy of Agricultural Sciences (CAAS), National Key Facility for Crop Gene Resources and Genetic Improvement, Key Laboratory of Biology and Genetic Improvement of Triticeae Crops, Ministry of Agriculture, Beijing, China; ^3^State Key Laboratory of Crop Stress Biology for Arid Areas, College of Plant Protection, Northwest A&F University, Shaanxi, China

**Keywords:** C2 domain protein, drought tolerance, salt tolerance, *Arabidopsis*, soybean

## Abstract

Plant C2 domain proteins play essential biological functions in numerous plants. In this study, 180 soybean C2 domain genes were identified by screening. Phylogenetic relationship analysis revealed that C2 domain genes fell into three distinct groups with diverged gene structure and conserved functional domain. Chromosomal location analysis indicated that C2 domain genes mapped to 20 chromosomes. The transcript profiles based on RNA-seq data showed that *GmC2-58*, *GmC2-88*, and *GmC2-148* had higher levels of expression under salt, drought, and abscisic acid (ABA) treatments. *GmC2-148*, encoding a cell membrane-localized protein, had the highest level of response to various treatments according to real-time quantitative polymerase chain reaction (RT-qPCR) analysis. Under salt and drought stresses, the soybean plants with *GmC2-148* transgenic hairy roots showed delayed leaf rolling, a higher content of proline (Pro), and lower contents of H_2_O_2_, O^2–^ and malondialdehyde (MDA) compared to those of the empty vector (EV) plants. The results of transgenic *Arabidopsis* in salt and drought treatments were consistent with those in soybean treatments. In addition, the soybean plants with *GmC2-148* transgenic hairy roots increased transcript levels of several abiotic stress-related marker genes, including *COR47*, *NCDE3*, *NAC11*, *WRKY13, DREB2A*, *MYB84*, *bZIP44*, and *KIN1* which resulted in enhanced abiotic stress tolerance in soybean. These results indicate that C2 domain genes are involved in response to salt and drought stresses, and this study provides a genome-wide analysis of the C2 domain family in soybean.

## Introduction

Environmental stresses caused by such factors as salt and drought significantly affect plant growth and metabolic processes, threaten the survival of plants, and often lead to reduction in yield (Bohnert et al., 1995). In response to stress, plants have developed suitable transduction pathways and a variety of tools to resist cell damage caused by stress (Chenu et al., 2008).

Many gene families related to abiotic stress have been identified, including regulatory factors and regulatory proteins, such as NAC, AP2/ERF, MYB, MYC transcription factors (Zhao et al., 2017), and C2 domain proteins, that are involved in plant signaling pathways (Liu et al., 2019). Many intracellular and extracellular proteins are partially or entirely composed of protein modules. The C2 domain is a typical intracellular protein module (Chapman et al., 1996). Being a Ca^2+^-dependent membrane targeting domain, this domain shows Ca^2+^-dependent phospholipid binding activity and a higher sensitivity to salt and drought stresses (Banci et al., 2002). The Ca^2+^ binding sites of most C2 domain proteins are associated with the presence of five aspartic acid residues, which are highly conserved in plants and animals (Lopez-Nicolas et al., 2006).

It is common knowledge that Ca^2+^ is widespread across in plants as a subordinate messenger and is involved in plant environmental response, growth, development and hormonal signaling (Cho et al., 2016). In recent years, most research have indicated that Ca^2+^ signaling takes part in plant answers to abioticstresses, including salt and drought stresses (Pak Dek et al., 2020). When Ca^2+^ binding proteins are bound to Ca^2+^, these proteins help transmit a signal to a downstream signaling pathway to induce expression of stress response genes. Four main Ca^2+^-binding proteins have been identified in the reaction of plants to modification in the exterior surroundings (Molz et al., 1996).

In plants, C2 domain genes play important roles, but they vary from species to species (Corbalan-Garcia et al., 2003). It is reported that *Cmpp16-1*, a C2 domain gene identified in pumpkin, was associated with RNA delivery (Ramirez-Ortega et al., 2014). Another C2 domain gene, *OsERG1*, was identified from rice suspension-cultured cells treated with fungal elicitors and reportedly respond to abiotic stresses and membrane translocation in a Ca^2+^-dependent manner (Kang et al., 2013). In recent years, the proteins of C2 domain family have been recognized as innovative monitoring proteins involved in a variety of abiotic stresses in plants. Three C2 domain proteins, containing the barley HvC2d1 (Ouelhadj et al., 2006), mung bean VrPLC3 (Wang, 2002; Vasseur et al., 2014), and *Arabidopsis* SYT1 proteins (Yamazaki et al., 2008) were all involved in stresses reaction through a Ca^2+^-dependent signaling-pathway. In pepper, a gene encoding a C2 domain protein was considerably upregulated under abiotic stresses, containing salt stress (Kim et al., 2008). In transgenic *Arabidopsis*, overexpression of *OsSMCP1* augmented tolerance to stresses compared to the control group (Yokotani et al., 2009). Together these are some of the studies in the literature that demonstrate the significant roles of C2 domain genes in numerous biological processes, so it is of great importance to study the functions of the C2 domain genes in plants (Nalefski and Falke, 1996). Although the C2 domain family has been analyzed in several plant species, no systematic investigations have been conducted in soybean (*Glycine max*).

Soybean is both a major global cereal crop and an essential tool in plant research. Particularly for countries such as China, soybean is a vital economic crop for cooking oil, soy products, soy sauce and protein extracts (Cerezini et al., 2020). However, in recent years shortages of water resources have exacerbated drought stress in plants which negatively affect crop cultivation and production in China (Kim et al., 2020). Thus, researchers have been employing molecular biology and genetic approaches to improve soybean yield and quality by enhancing its stress tolerance. Recently, with the expansion of biotechnology, it is effective to predict the gene function of a large gene family through genome-wide identification and analysis (Agarwal et al., 2019). Thus, we endeavored to systematically study the C2 domain family in soybean, given our current knowledge of its importance in plant stress tolerance and the availability of genomic information.

In this study, we identified 180 soybean C2 domain genes and analyzed their phylogenetic relationship, chromosomal distributions, motif compositions, *cis*-elements, and expression patterns in various treatments. To establish the role of the soybean C2 domain genes in response to stresses, we evaluated their response to various treatments, which include salt and drought stresses, as well as ABA treatment. Using real-time quantitative polymerase chain reaction (RT-qPCR) analysis, we revealed a gene, *GmC2-148*, with the strongest responses to these treatments. We simultaneously verified the resistance of *GmC2-148* to abiotic stresses in soybean and *Arabidopsis* demonstrated that G*mC2-148* possibly will improve acceptance to salt and drought stresses. The results presented in this report will expand our knowledge of the C2 domain family in soybean.

## Materials and Methods

### Identification and Sequence Analysis of C2 Domain Genes

Protein sequences of the entire C2 domain in soybean and *Arabidopsis* were attained from Phytozome^1^. We downloaded the Hidden Markov model (HMM) profiles of the C2 domain (PF00168) to search C2 domain genes in the soybean dataset using the software HMMER 3.0 (HMMER^2^) with a threshold of e-value < e^–5^. The CDD^3^, Pfam^4^, and SMART^5^ databases were used to determine the conservative C2 domain genes. Calculation biophysical properties of C2 domain genes using the online server tool ExPASy^6^.

### Phylogenetic Relationship, Chromosomal Distribution, and Gene Duplication of C2 Domain Genes

Multiple sequence alignment of the C2 domain genes from soybean and *Arabidopsis* was performed by ClustalW2^7^. Using pairwise distances and the neighbor-joining (NJ) algorithm, we constructed a phylogenetic tree by MEGA7.0 with 1,000 bootstrap. All C2 domain genes were separately mapped on the 20 soybean chromosomes based on information from the Phytozome database. Multiple collinear scanning toolkits (MCScanX^8^) with the default parameters were used to analyze the gene replication events.

### Gene Structure, Motif Composition and *cis*-Acting Element, Tissue-Specific Expression Patterns and *cis*-Acting Element Analyses of C2 Domain Genes

The gene structure map is generated from the online website^9^. Motif information was obtained by MEME^10^. Then we imported the images generated by the online websites into TBtools^11^ to generate a cluster composition (Chen et al., 2020). As for the analysis of tissue expression pattern, we uploaded the information of C2 domain genes to the online website soybase^12^ and drew the heat map of gene expression patterns in different tissues by TBtools. Another online tool, PLACE^13^, was applied to predict the soybean C2 domain gene promoters. We selected the 1,500 bp upstream from the ATG start codon of *GmC2-58*, *GmC2-88*, and *GmC2-148* to analyze their *cis*-acting elements. The genes were submitted to PLACE to identify the *cis*-acting elements that are related to abiotic stresses.

### RNA Extraction

We cultivated 10 “Williams 82” soybean seeds in a 12 cm × 12 cm pot filled with vermiculite and soil at a 1:3 ratio and established a total of 18 pots for all treatments. Fresh soybean leaves of 10-day-old plants were obtained to extract RNA after individuals were exposed to the various treatments. For the drought treatment, soybean seedlings were dried on filter paper before obtaining 0.1 g of leaf tissue at different time periods 0, 1, 2, 4, 8, 12, and 24 h. We pulled the soybean seedlings out of the soil and put them in a cup for another treatment. The salt, ABA and BR treatments of soybean seedlings contained 200 mM NaCl, 100 μM ABA and 100 μM BR, respectively. Additionally, we established a high temperature treatment by putting soybean seedlings in an oven set at 45°C and a low temperature treatment by putting soybean seedlings in a refrigerator at 4°C. Sampling of 0.1 g of leaf tissue occurred at the same time period as sampling occurred in the drought treatment.

The RNA of soybean roots treated with salt and dry pot culture was extracted and reverse transcribed to analyze the expression level of salt and drought related marker genes. We used the RNA prep plant kit produced by (TianGen, China) and the cDNA synthesized kit (Transgen, China) to obtain cDNA for follow-up experiments. The concentration of cDNA was diluted to about 200 ng/ul.

### Analyses of C2 Domain Gene Expression Patterns in Various Treatments

The expression levels of three C2 domain genes, *GmC2-58*, *GmC2-88*, and *GmC2-148* exposed to the various treatments were analyzed in soybean. RT-qPCR was accompanied with an ABI7500 real-time PCR system (ABI, United States) using TransStart Top Green qPCR SuperMix (Transgen, China). The data of each gene expression is calculated by using the method of 2^−ΔΔCt^ method. Expression of *GmC2-58*, *GmC2-88*, and *GmC2-148* were measured relative to the control (0 h). Using actin gene as internal control, the template cDNA was normalized. The total volume containing 2 × Taq PCR Master Mix was 20 μl (TianGen, China). The primer sequences used in this paper are all shown in the Supplementary Figure 2.

### Gene Ontology Annotation

According to Conesa and Gotz (2008), we used the on line website Blast2GO^14^ to annotate and analyze the C2 domain family.

### Subcellular Localization

We constructed an expression vector with green fluorescent protein (GFP) marker for subsequent subcellular localization analysis as described earlier (Cui et al., 2019). The coding sequence length (CDS) of *GmC2-148* was specifically amplified by PCR, then the correctly sequenced plasmid was amplified with the splice primer with GFP, and transformed in *Escherichia coli* to extract the fusion plasmid of *GmC2-148*-GFP. The recombinant plasmid of *GmC2-148*-GFP was transformed into *Arabidopsis* protoplasts using PEG4000-mediated method (Momota et al., 2020). After incubating in the dark at 23°C for 18–24 h, the protoplasts were detected by laser confocal microscope (Carl Zeiss LSM 700, Germany).

### *Agrobacterium rhizogenes*-Mediated Transformation of Soybean Hairy Roots

To generate the soybean plants with *GmC2-148* transgenic hairy roots, the *GmC2-148*-3301 recombinant vector and empty vector (EV) were introduced into *Agrobacterium* competent cells of strain K599 and then transformed into soybean hairy roots by *A. rhizogenes*-mediated transformation as described earlier (Kereszt et al., 2007). We covered each infected soybean plant with a transparent plastic cup that was 25cm high and 8cm wide (at the bottom) to maintain the temperature at 25°C and air humidity at 70%, and the gaps in cups were filled with soil. After 2 weeks, hairy roots were observed at the infected site on the outside of the plastic cup, and then the main root 1 cm below the infected site was removed. Four seedlings were transplanted into a pot, totaling 24 pots containing mixed soil (1:1 vermiculite/humus) and then cultured at 25°C and a 16 h light/8 h dark photoperiod in a greenhouse.

### Transformation of *Arabidopsis* and Propagation of Positive Seedlings

We ligated the CDS sequence of *GmC2-148* to vector pCAMBIA1302 to construct the transgenic vector of *GmC2-148* in *Arabidopsis*. After being transformed into *E. coli* and sequenced rightly, the plasmid was extracted and transformed by *Agrobacterium* competent cell GV3101. The OD600 value of *Arabidopsis* infection solution was adjusted to 0.8, then the inflorescence of *Arabidopsis* was soaked in the infection solution for 3 min, repeated twice, and the second infection was carried out 1 week after infection. Two weeks after infection, DNA was extracted from the leaves of *Arabidopsis* for positive detection, and RNA was extracted to detect the expression level. After harvesting the seeds of *Arabidopsis* positive lines with high expression level, the seedlings were screened on the medium of hygromycin, then planted and cultured to T2 generation for experiment.

### Salt and Drought Tolerance Analyses

For the salt stress assay of soybean, EV and the soybean plants with *GmC2-148* transgenic hairy roots were treated with 250 mM NaCl solution for 7 days. For the drought stress assay, EV and the soybean plants with *GmC2-148* transgenic hairy roots were grown while watering was withheld for 7 days. After the drought treatment, we re-watered soybean plants for 3 days and then observed their growth. A control was also established for both treatments where plants were watered with 250 ml of water per pot three times a week.

As for experiment on salt treatment of *Arabidopsis*, wild type (WT) and *GmC2-148* transgenic plants were treated with 250 mM NaCl solution for 7 days. During the drought treatment, *Arabidopsis* were grown while watering was withheld for 10 days. In the *Arabidopsis* root length experiment, we used 6 and 9% (w/v) polyethylene glycol (PEG) to simulate drought, and treated *Arabidopsis* with 90 and 110 mM NaCl, 10 and 12 μM ABA to observe the root length phenotype.

### Measurements of Malondialdehyde, Proline, H_2_O_2_, and O^2–^ Contents

During this study, we measured malondialdehyde (MDA), Pro, H_2_O_2_, and O^2^*^–^* contents from soybean leaves after the salt and drought stress treatments. For each physiological index, we sampled leaves from three individuals. Contents of MDA, Pro, H_2_O_2_, and O^2^*^–^* were measured 0.1 g from leaf samples follow the instructions of the kit (Solarbio, China). Determination of absorbance value by Varioskan LUX multimode enzyme labeling instrument (Thermo Fisher Scientific, United States).

### Measurements of Chlorophyll Content and Electrical Conductivity

To determine the content of chlorophyll, took 0.1 g from soybean leaves and cut them up, placed in a centrifuge tube, and filled up to 10 ml with 80% acetone solution before placing the tube in the dark for 24 h. The chlorophyll extract’s reflectance was then measured at wavelengths of 665 and 649 nm to determine the chlorophyll content. An 80% acetone solution was used as the control.

To prepare materials for measurement of electrical conductivity, plant leaves of the same size were cleaned with paper and any water remaining on their surfaces was absorbed with filter paper. Then we cut the leaves into pieces of the same size, mixed them evenly and took 0.1 g of the cut pieces to soak them in 10 ml water for 12 h. Next, we measured the conductivity R1 of the solution of steeped leaf sample with a conductivity meter then putting samples in boiling water for 30 min and then cooling them to 25°C. We then measured R2 to calculate relative conductivity of samples based on the following equation: *R*1/*R*2 × 100%.

### Nitrogen Blue Tetrazole and Trypan Blue Staining

Individual intact leaf samples from salt and drought stressed soybean and a control soybean group of plants were used in two staining procedures to observe the degree of damage to the leaves. For Nitrogen Blue Tetrazole (NBT) staining, the cut leaves were soaked in NBT solution for 14 h, and then immersed in 75% ethanol for decoloration until the sample became transparent. For trypan blue staining, we first prepared 10 ml of 0.4% trypan blue solution that was filtered and sterilized before adding 90 ml 1 × PBS solution. The leaves were placed in 100 ml of the mixed solution and kept at 37°C in the dark for 24 h. Dyed leaves were then added to a 1:3 ethanol:glacial acetic acid solution to decolorize leaves at 37°C in the dark for 24 h until the samples became transparent.

### Measurements of Relative Water Content

Relative water content was determined using the following equation: relative water content (%) = (fresh weight − dry weight)/(saturated fresh weight − dry weight) × 100%. Soybean leaves were cut into pieces and then weighed to obtain fresh weight. They were subsequently soaked for a few hours until the leaves were saturated with water. Then their leaf surfaces were dried with absorbent paper and the leaves were weighed immediately to obtain their saturated fresh weight. The same leaves were then oven-dried at about 70–80°C for 2 days until they reached a constant weight. Dried samples were placed them on a table at 25°C for 2 h before measuring the dry weight (Li et al., 2020).

## Results

### Identification and Phylogenetic Analysis of C2 Domain Genes in the Soybean Genome

**FIGURE 1:**
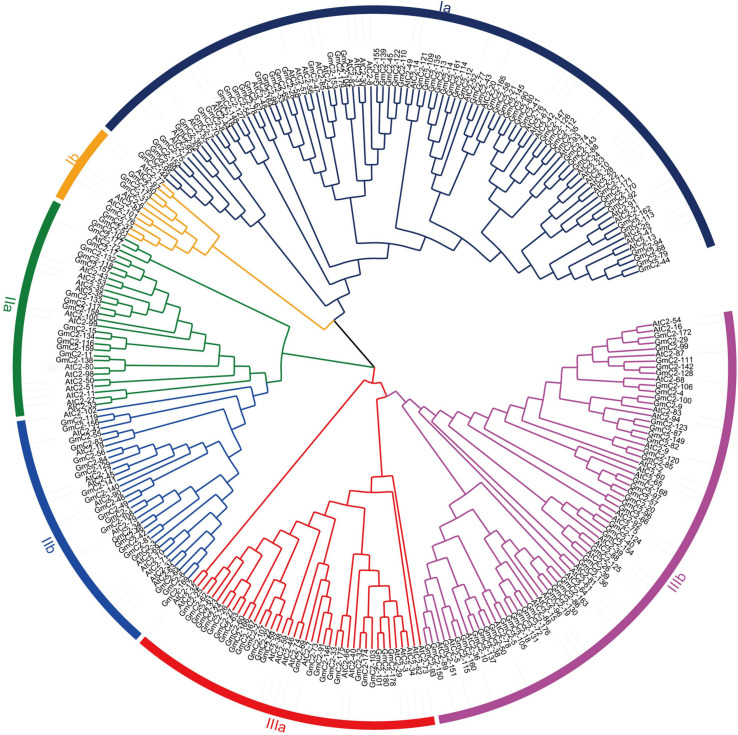
Phylogenetic relationship of C2 domain genes between soybean and *Arabidopsis*. The phylogenetic tree was generated by comparison of the C2 domain amino acid sequences in MEGA 7. The neighbor-joining method was used and the bootstrap value was set to 1,000. All the genes were divided into three groups, I, II, and III.

We found 196 potential C2 domain genes in the soybean genome (*G. max* Wm82.a2.v1). After screening from SMART and Pfam databases, we identified 180 C2 domain family members in soybean. The statistical results exhibited that the amino acid sequence length of the 180 C2 domain genes varied from the shortest of 128 amino acids to the longest of 1,181 amino acids, and the length of CDS varied from 384 to 3,543. The isoelectric point (p*I*) varied from 4.8 to 5.32, and the molecular weights (MW) ranged from 27.9 to 292.5 kDa (Supplementary Table 1). We constructed an unrooted NJ phylogenetic tree to analyze the evolutionary relationships in the C2 domain family. The tree, constructed using the 180 soybean C2 domain genes and included 102 *Arabidopsis* C2 domain genes, classified the proteins into three major groups (I–III) and six subgroups (Ia, Ib, IIa, IIb, IIIa, and IIIb) according to their genetic relationships (Figure 1).

### Chromosomal Distribution and Gene Duplication of C2 Domain Genes

**FIGURE 2:**
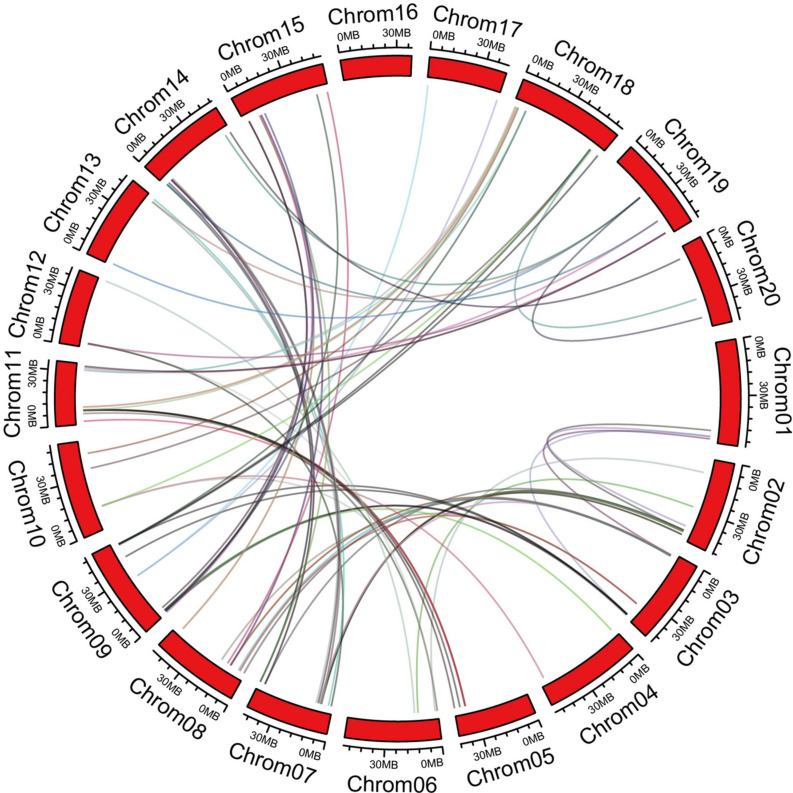
Orthologous relationships of 180 C2 domain genes in soybean. Numbers along each chromosome box indicate sequence lengths in mega bases. Lines between two chromosomes represent the syntonic relationships.

The chromosome information of C2 domain genes was extracted and the chromosomal positions indicated that the 180 C2 domain genes were unequally distributed in 20 soybean chromosomes (Supplementary Figure 1). Each chromosome contained ≥1 C2 domain gene. Chromosome 7 contained the highest number of C2 domain genes, while chromosomes11, 16, and 20 contained the lowest numbers of C2 domain genes. In order to disclose the extension mechanism of the C2 domain family, all soybean replication data files are filtered by MCScanX (Figure 2). Fragmented duplicated genes were the most frequently found on chromosome 8, followed by chromosome 7, whereas there were no pairs of duplicated fragmented genes on chromosome 16.

### Gene Structure and Motif Composition of Soybean C2 Domain Genes

**FIGURE 3:**
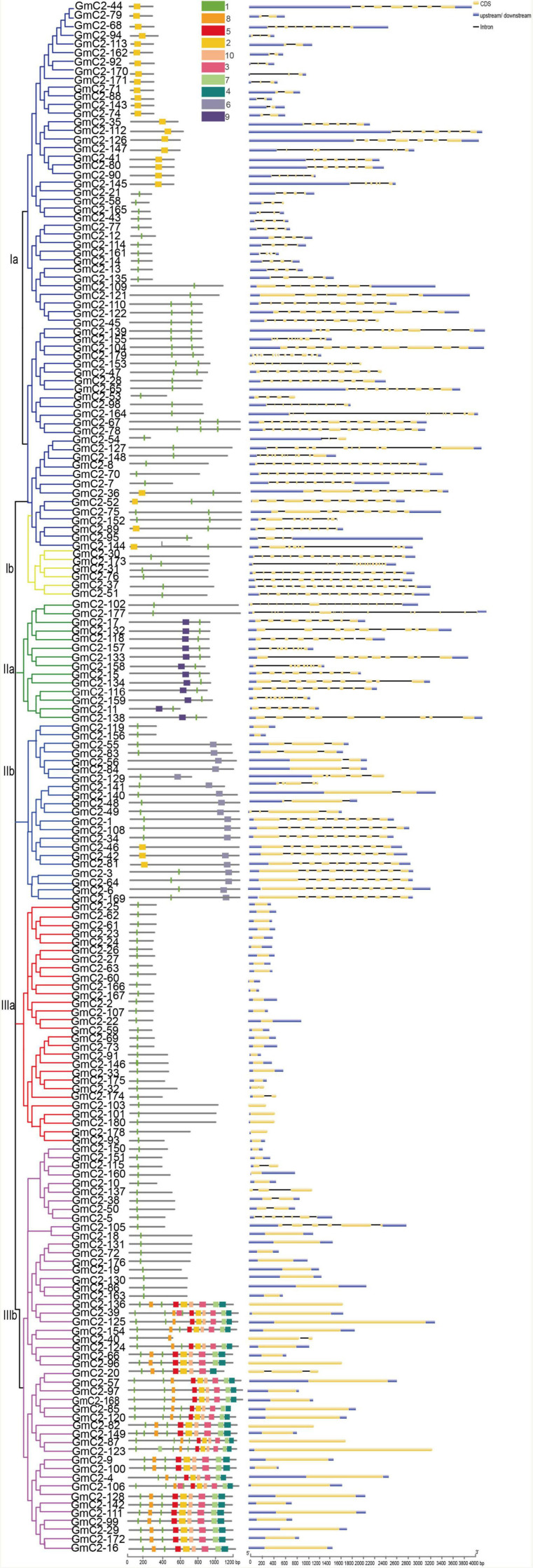
Motif and gene structure analyses of 180 C2 domain genes. The gene sequences of 180 C2 domain genes were analyzed with online tools. The maximum number for motif is 10. There are great differences in the number of motifs and intron-exons in different genes.

To further understand the evolutionary relationship of the 180 C2 domain genes, we identified structural features and motifs of the C2 domain genes from soybean, including the locations of exons and introns and conserved motifs (Figure 3). According to our statistics, the number of introns in C2 domain family genes is varied from 1 to 18 and the number of introns considerably varied among the C2 domain genes. Most C2 domain genes in group Ia and IIa contain 8 introns, *GmC2-70* in group Ia has the largest number of introns, containing 18 introns, *GmC2-102* in group IIa has the largest number of introns, containing 17 introns. Most C2 domain genes in group Ib contain 15 introns, while most C2 domain genes in group IIb contain 9 introns. Most of the genes in group IIIa and IIIb have no introns. Also, the number of motifs contained in different genes varied greatly. Among the C2 domain genes, 44 had 1 motif, 34 had 2 motifs and 32 had 3 motifs, respectively accounting for 24.5, 18.8, and 17.7% of the total number of C2 domain genes. As for motif, group Ia containing motif 2 or 7, C2 domain genes in group Ib containing motif 7, most C2 domain genes in group IIa containing motif 7 and 9, group IIb containing motif 6 and 7, group IIIa and some C2 domain genes in group IIIb contained one motif 7, and the rest of the group IIIb genes contained other eight different motifs. Ten motifs are shown in the table (Table 1).

**TABLE 1:** Information about 10 motifs of C2 domain family.

Motifs	Best possible match	Size
1	TNPVWNEKFMF	11
2	TTDAYCVAKYGQKWVRTRTIIDSFAPRWNEQYTWEVFDPCTVI TIGVFDN	50
3	QIDNLRYQAMNIVAARLGRAEPPLRKEVVEYMLDVDSHMW SMRRSKANFF	50
4	DIVRMRYDRLRSIAGRIQTVVGDLATQGERLQSLLSWRDPRATA LFVIFC	50
5	HMRICLEGGYHVLDESTHYSSDLRPTAKQLWKSS IGVLELG	41
6	RRFMIYVHSKGMIVDDEYVILGSANINQRSMEGTRDTEIAMG AYQPHHTW	50
7	FLIGIWNFRWRPRHPPHMDTRLSHADAAHPDELD EEFDTFP	41
8	KGELMLAVWMGTQADEAFPEAWHSDAATV	29
9	TSSNYRPHIGWMYGAQMVAFNMQGHGKSLWYMQGMFRA NGGCGYVKKPAF	50
10	DSKIGKVRIRLSTLETDRVYTHSYPLLVLHP	31

### Transcript Profiles of Soybean C2 Domain Genes in Response to Multiple Abiotic Stresses

**FIGURE 4:**
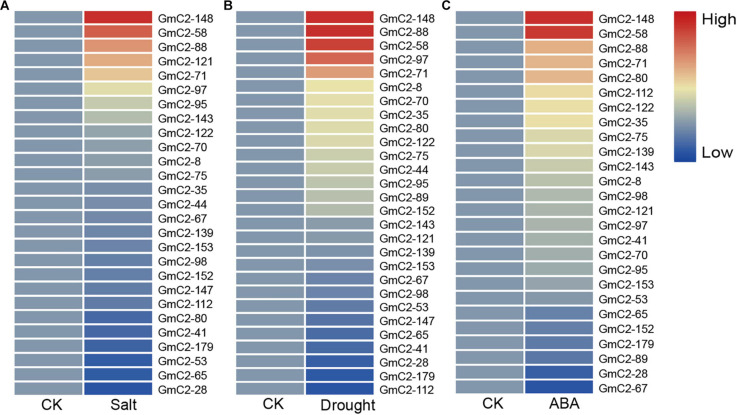
RNA-seq analyses of the C2 domain genes from group I under various treatments. **(A)** Salt treatment transcript profiles, **(B)** Drought treatment transcript profiles, **(C)** ABA treatment transcript profiles. The heat map was produced by TBtools software, and different colors on the scale bar represent different transcript levels.

The date of C2 domain genes differentially expressed under NaCl, drought and ABA treatments are from the RNA-seq results of our laboratory, which have been used in the paper by Shi et al. This RNA-Seq date comes from 10-day-old soybean seedlings that were subjected to NaCl, drought and ABA treatments (Shi et al., 2018). To study the soybean C2 domain genes in response to these treatments, we investigated the transcript profiles of them. Because under abiotic stress, compared with the other two groups, group I had the largest number of gene responses, implying that C2 domain genes in group I are more likely involved in soybean reaction to abiotic stress. Hence, we used a heat map to visually analyze the expression patterns of these potential stress-related genes in group I. The results presented that most of the soybean C2 domain genes in group I showed varying degrees of response to stress. What is more, we found that *GmC2-58*, *GmC2-88*, and *GmC2-148* all exhibited greater levels of expression in response to the stress treatments, so then we selected these three genes for further investigation (Figure 4).

### Tissue-Specific Expression Patterns of C2 Domain Genes

**FIGURE 5:**
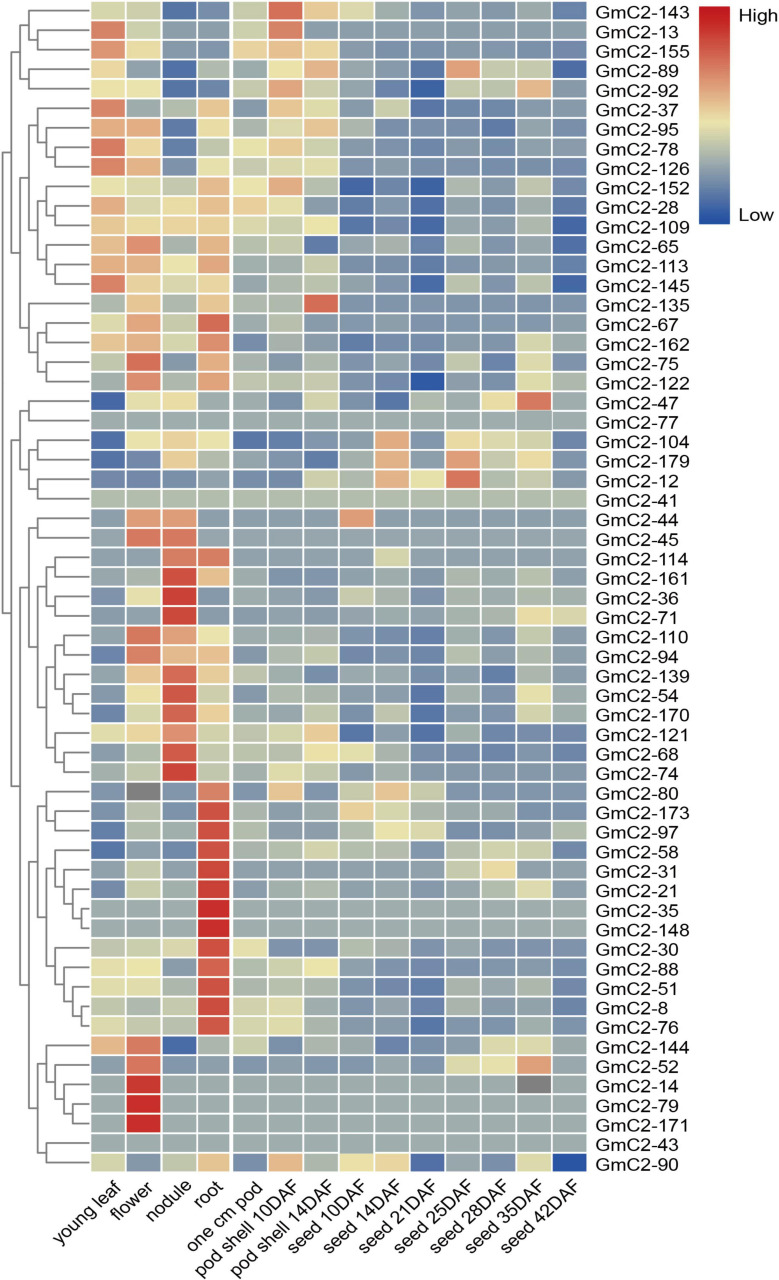
Tissue-specific heat map of expression profiles (in log_10_-based FPKM) of C2 domain genes in group I. The expression levels of different transcripts are represented by the color bar; red indicates high expression levels, while blue indicates low expression levels.

To comprehend the tissue-specific expression patterns of soybean C2 domain genes, their expression profiles in different tissues were obtained from the soybean database. We explored profiles of the C2 domain genes from group I in various tissues because they showed the highest responses in stressful abiotic conditions, and the hierarchical grouping of tissue-specific expression patterns could reflect the expression of these genes under normal conditions (Figure 5). Expression data displayed most soybean C2 domain genes showed diverse transcript levels in various tissues. In particular, *GmC2-58*, *GmC2-88*, and *GmC2-148* all produced higher expression levels in roots, the important vegetative organ that functions to absorb water and inorganic salts from the soil, suggesting that the selected genes may have an important role in plant growth. The relevant data expressed by the organization are shown in the Supplementary Table 6.

### Promoter Regions of C2 Domain Genes Contain Various Stress Response Elements

In order to further elucidate the regulatory mechanism of C2 domain genes, we isolated the promoter regions of three selected genes *GmC2-58*, *GmC2-88*, and *GmC2-148*. The salt-responsive element GT1 and ABA-responsive element ABRE4 are two types of *cis*-elements associated to abiotic stress (Yang et al., 2020). We found these two elements, as well as the GA-responsive element W-BOX (Dhatterwal et al., 2019), drought-responsive elements MYB and DPBF (Carre and Kim, 2002), and wound-responsive element MYC (Ciznadija et al., 2009) were located within our three selected genes. These results further supported that C2 domain genes may confer abiotic stress tolerance to soybean.

### Gene Ontology Annotation

To better understand the possible biological function of C2 domain genes, Blast2GO software were used to performed the GO ontology annotation (Supplementary Figure 3). According to the GO annotation results, the C2 domain proteins were annotated in various biological processes, including response to stress, negative regulation of cell death, intracellular signal transduction, phosphatidylcholine metabolic process, and lipid catabolic process. There were 134 C2 domain proteins were predicted to be related to molecular functions, such as calcium ion binding, calcium-dependent phospholipid binding, lipid binding, N-acylphosphatidylethanolamine-specific phospholipase D activity, signal transducer activity and GTPase activator activity. Finally, 105 C2 domain proteins were belonged to cellular components, which were located to integral component of membrane, membrane, intracellular, endoplasmic reticulum and plasma membrane. Through the GO annotation of C2 domain family, it is found that most of C2 domain genes respond to stress and depend on calcium phospholipid binding. This analysis is helpful for us to further understand and analyze C2 domain family.

### Responses of Soybean C2 Domain Genes to Various Treatments

**FIGURE 6:**
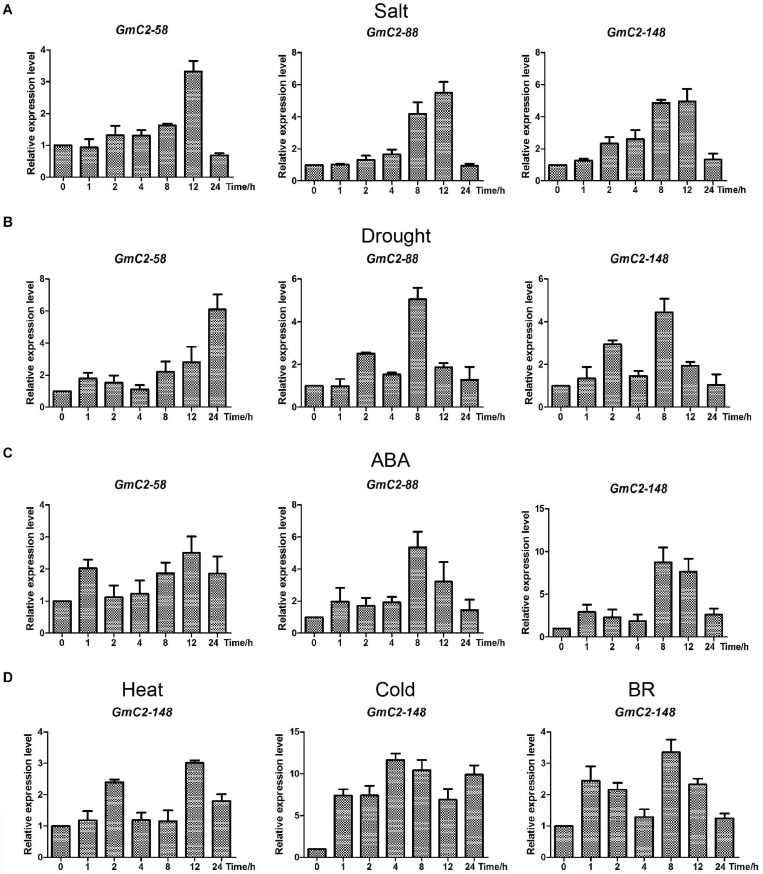
Expression patterns obtained by RT-qPCR of the three selected C2 domain genes from soybean exposed to salt, drought, and ABA treatments. **(A)** Durations of salt treatment exposure were 0, 1, 2, 4, 8, 12, and 24 h. **(B)** Drought treatment durations were 0, 1, 2, 4, 8, 12, and 24 h. **(C)** ABA-treated leaves were sampled at the same times as the samples for the salt and drought treatments. **(D)** Expression patterns of *GmC2-148* under heat, cold and BR treatments by RT-qPCR. The vertical coordinates are fold changes, and the horizontal ordinates are treatment times. The actin gene was used as an internal reference. Data are means of three biological repeats ± SD.

To further illuminate salt and drought stress responses of C2 domain genes, *GmC2-58*, *GmC2-88*, and *GmC2-148* were investigated by RT-qPCR from soybean seedlings exposed to salt, drought and ABA treatments (Figure 6). Among the three genes, *GmC2-148* obtained the highest expression levels in the various treatments, thus it was selected for subsequent experiments. In order to verify that *GmC2-148* could respond to a variety of treatments, we performed RT-qPCR on soybean exposed to additional treatments of high temperature, low temperature and external spraying of BR (Figure 6). In all of these additional treatments, *GmC2-148*, again showed high levels of expression, further supporting that C2 domain genes respond to various treatments.

### Subcellular Localization

**FIGURE 7:**
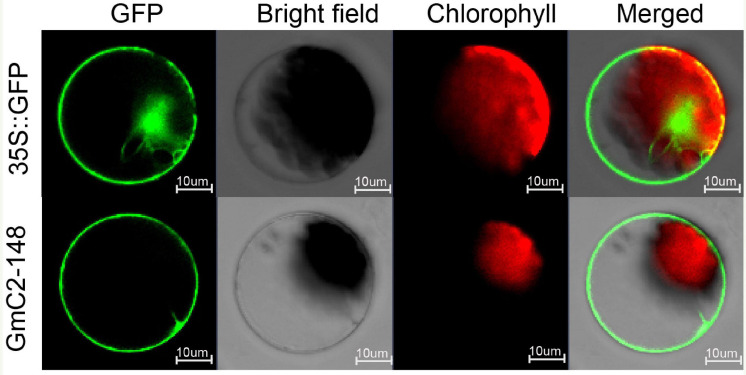
Localization of C2 domain protein *GmC2-148* in *Arabidopsis* protoplasts. The images of the construct transferred into *Arabidopsis* protoplasts were observed under a laser scanning confocal microscope. Bar = 10 μm.

Through online website analysis, we found that *GmC2-148* is a transmembrane protein with typical C2 domain (Supplementary Figures 4,5). To scrutinize the subcellular localization of *GmC2-148*, we prepared protoplasts from *Arabidopsis* and carried out subcellular localization experiments. The 16318hGFP was used as a control, GFP fluorescence was observed throughout cells. Meanwhile the *GmC2-148*-GFP fusion protein was mainly localized in cell membrane indicating that *GmC2-148* was located in the cytomembrane (Figure 7).

### The Soybean Plants With GmC2-148 Transgenic Hairy Roots Significantly Improved Salt Tolerance in Soybean Hairy Roots

**FIGURE 8:**
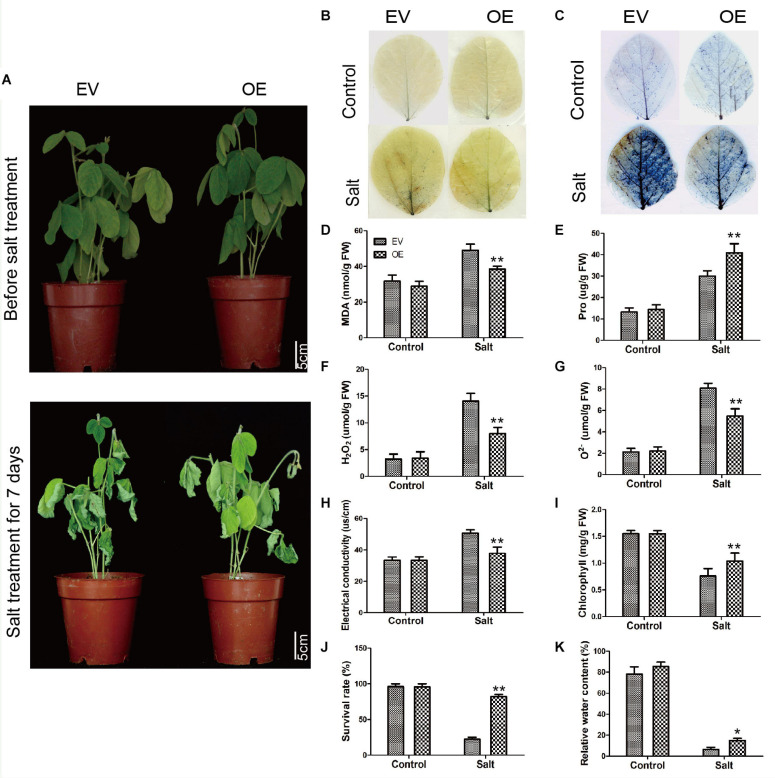
Salt stress analyses of EV and the soybean plants with *GmC2-148* transgenic hairy roots. **(A)** Phenotypes of EV and the soybean plants with *GmC2-148* transgenic hairy roots under control and salt treatments. **(B)** NBT and **(C)** Trypan blue staining of EV and the soybean plants with *GmC2-148* transgenic hairy roots under control and salt treatments; dead cells stain, while living cells do not stain. **(D)** MDA content, **(E)** Pro content, **(F)** H_2_O_2_ content, **(G)** O^2−^ content, **(H)** electrical conductivity, **(I)** chlorophyll content, **(J)** survival rate, and **(K)** relative water content of EV and the soybean plants with *GmC2-148* transgenic hairy roots under normal and salt conditions. Data are means ± SD obtained from three biological replicates. Significant differences determined by ANOVA at *p < 0.05 and **p < 0.01.

To further explore the biological function of the C2 domain gene *GmC2-148* in salt response, we carried out salt treatment on EV and the soybean plants with *GmC2-148* transgenic hairy roots to observe the phenotype and mark data statistics. When under normal growth conditions, no significant differences were observed between the EV and the soybean plants with *GmC2-148* transgenic hairy roots. In contrast, apparent differences were found in the growth and physiology between the EV and the soybean plants with *GmC2-148* transgenic hairy roots after salt treatment for several days. Compared with the control group, the salt treatment group clearly exhibited curled and yellow leaves and a lower number of green leaves. Additional physiological data differed between control and treatment plants. The soybean plants with *GmC2-148* transgenic hairy roots leaves showed delayed leaf rolling, higher Pro content, and lower contents of MDA, H_2_O_2_, and O^2–^ compared with those of the EV control plants. The results of NBT and trypan blue-staining of leaves and other physiological indexes, such as electrical conductivity, chlorophyll content, survival rate, and relative water content, also showed that the resistance of the soybean plants with *GmC2-148* transgenic hairy roots under salt stress was significantly better than that of EV control plants (Figure 8). At the same time, the root length of the soybean plants with *GmC2-148* transgenic hairy roots after salt treatment was significantly longer than that of EV, because the root is an important organ for plants to absorb nutrients (Figure 10). The results of leaf staining and other physiological indicators also verify that the soybean plants with *GmC2-148* transgenic hairy roots may enhance soybean resistance to salt treatment.

### The Soybean Plants With *GmC2-148* Transgenic Hairy Roots Significantly Improved Drought Tolerance in Soybean Hairy Roots

**FIGURE 9:**
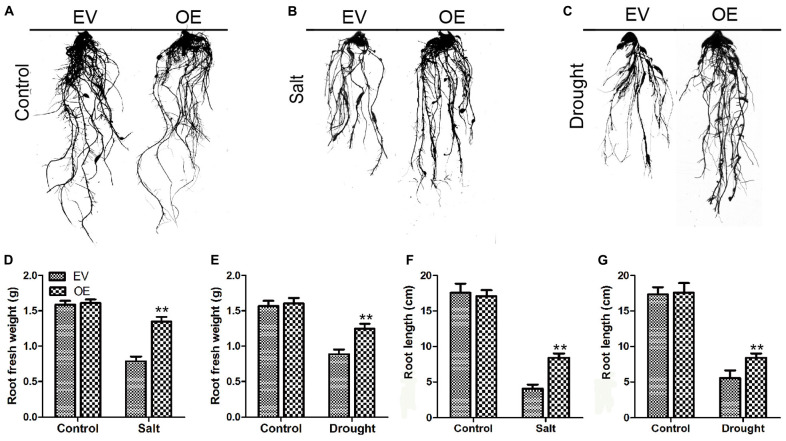
Root length investigations of EV and the soybean plants with *GmC2-148* transgenic hairy roots. **(A–C)** Root length pictures of EV and the soybean plants with *GmC2-148* transgenic hairy roots after salt and drought treatments. **(D)** Fresh weight of root after salt treatment. **(E)** Fresh weight of root after drought treatment. **(F)** Root length after salt treatment. **(G)** Root length after drought treatment. *p < 0.05 and **p < 0.01.

**FIGURE 10:**
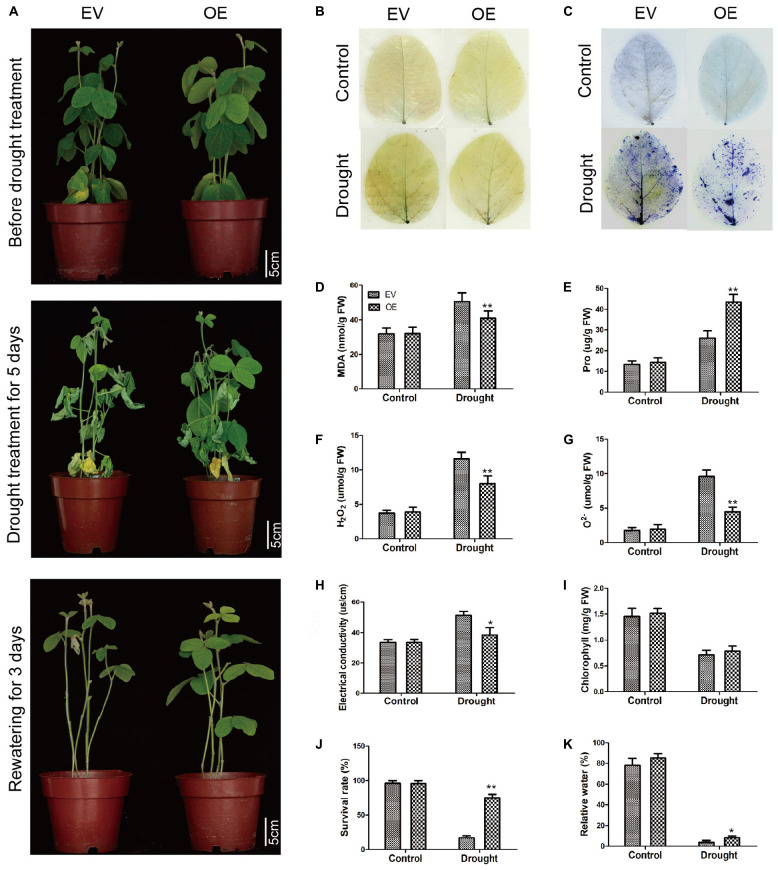
Drought stress investigations of EV and the soybean plants with *GmC2-148* transgenic hairy roots. **(A)** Phenotypes of EV and the soybean plants with *GmC2-148* transgenic hairy roots under control and drought treatments. **(B)** NBT and (C) Trypan blue staining of EV and the soybean plants with *GmC2-148* transgenic hairy roots under control and drought treatments; dead cells can be stained, while living cells cannot. **(D)** MDA content, **(E)** Pro content, **(F)** H_2_O_2_ content, **(G)** O^2−^ content, **(H)** electrical conductivity, **(I)** chlorophyll content, **(J)** survival rate, and **(K)** relative water content of EV and the soybean plants with *GmC2-148* transgenic hairy roots under normal and drought conditions. Data shown are means ± SD obtained from three biological replicates. Significant differences determined by ANOVA. *p < 0.05 and **p < 0.01.

To determine the roles of *GmC2-148* in drought response, a drought treatment was carried out on EV and the soybean plants with *GmC2-148* transgenic hairy roots. Similarly, we found that there was no noteworthy difference between EV and the soybean plants with *GmC2-148* transgenic hairy roots under normal growth conditions, but the leaf wilting and shedding of EV was more serious than that of the soybean plants with *GmC2-148* transgenic hairy roots under drought conditions, indicating that the soybean plants with *GmC2-148* transgenic hairy roots had stronger stress resistance under drought conditions. Similar to the salt stress treatment, the soybean plants with *GmC2-148* transgenic hairy roots accumulated more Pro and had lower MDA, H_2_O_2_, and O^2–^ contents (Figure 9). The results of NBT and trypan blue-staining of leaves and other physiological indicators measured after drought stress treatment verified that the soybean plants with *GmC2-148* transgenic hairy roots heightened soybean resistance to drought stress. Correspondingly, the root length of the soybean plants with *GmC2-148* transgenic hairy roots was considerably longer than that of EV after drought treatment (Figure 10).

### *GmC2-148* Conferred Salt and Drought Tolerance in *Arabidopsis*

**FIGURE 11:**
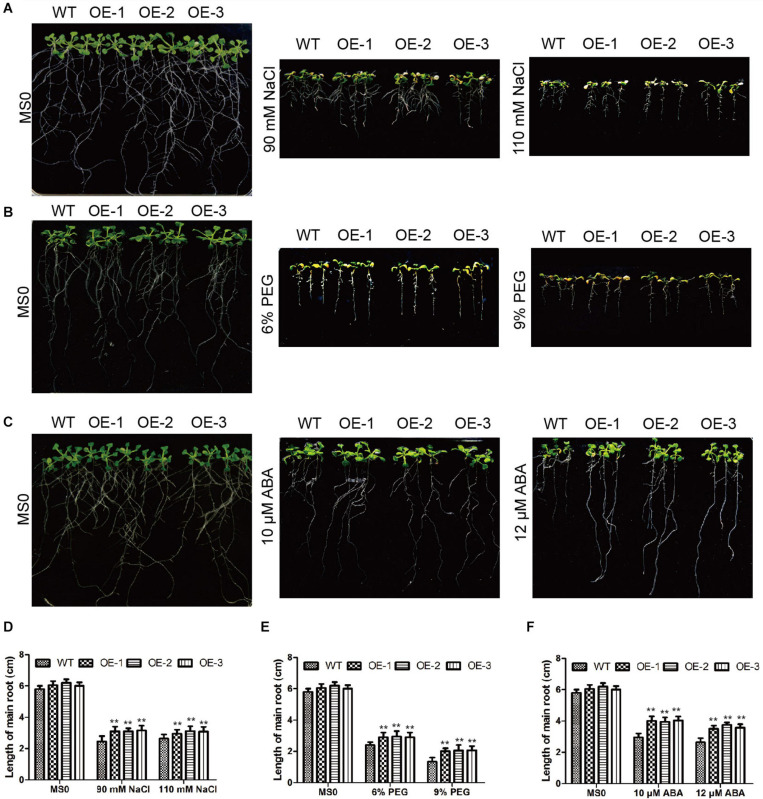
Root length investigations of EV and *GmC2-148* transgenic *Arabidopsis*. **(A–C)** Root length of transgenic plants and WT after NaCl, PEG and ABA treatments. **(D)** Statistics of root length data of transgenic plants and WT after salt treatment. **(E)** Root length of transgenic *Arabidopsis* and WT after PEG treatment. **(F)** Root length of transgenic *Arabidopsis* and WT after ABA treatment. *p < 0.05 and **p < 0.01.

**FIGURE 12:**
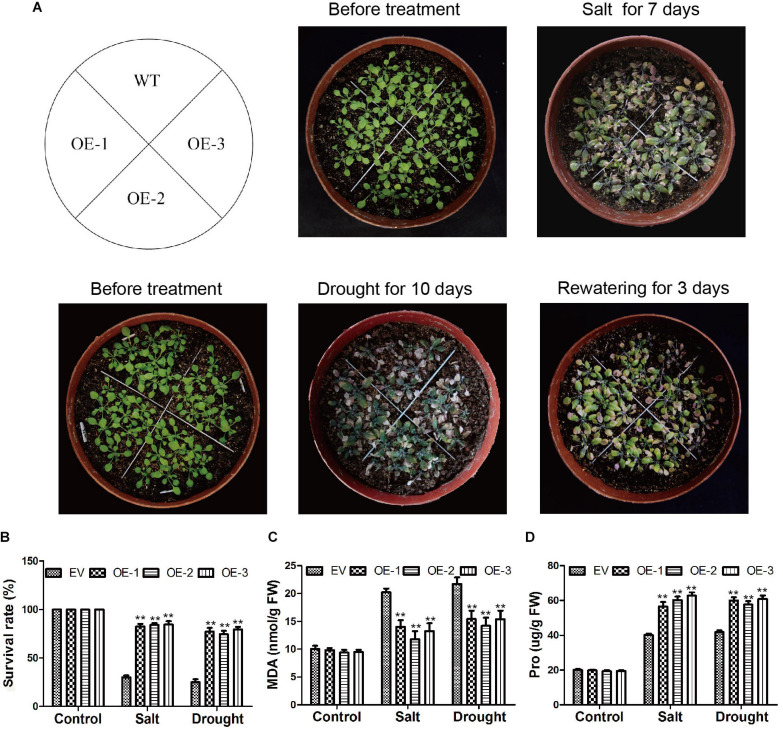
Phenotypic investigations of *Arabidopsis* after salt and drought treatments. **(A)** Phenotypes of EV and *GmC2-148* transgenic *Arabidopsis* plants under salt and drought treatments. **(B)** Statistics on the survival rate of transgenic plants and WT after salt and drought treatment. **(C)** MDA content of *Arabidopsis* after salt and drought treatments. **(D)** Pro content in transgenic *Arabidopsis* and WT after abiotic treatments.

In *Arabidopsis*, we also verified the *GmC2-148* transgenic plants resistance to abiotic stress. After *Arabidopsis* grown in 25°C and 70% humidity greenhouse for 7 days, and then treated them with salt and drought treatment respectively. In the root length experiment of *Arabidopsis* (Figure 11), the root length of transgenic plants treated with NaCl, PEG and ABA were longer than those of WT, indicating that *GmC2-148* improved the ability of *Arabidopsis* root to resist abiotic stress. As shown by Figure 12, the growth status of *GmC2-148* transgenic plants in *Arabidopsis* after adverse treatments was considerably better than that of WT, and the physiological indicators, survival rate, MDA and Pro also substantiated our results, showing that *GmC2-148* transgenic plants augmented the resistance of *Arabidopsis* to abiotic stress.

### The Soybean Plants With *GmC2-148* Transgenic Hairy Roots Increased Transcripts of Abiotic Stress-Related Marker Genes

**FIGURE 13:**
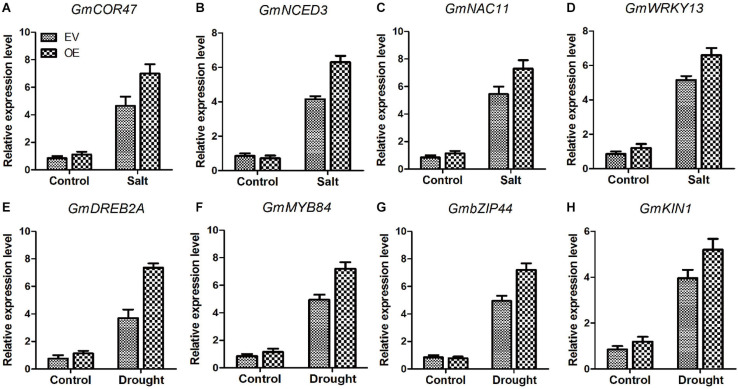
The soybean plants with *GmC2-148* transgenic hairy roots revealed greater levels of transcription of salt and drought-stress marker genes **(A–H)**. Two-week-old soybean seedlings were obtained for RT-qPCR analyses. The actin gene was used as an internal control. Data shown are means ± SD obtained from three biological replicates.

Several genes, *COR47* (Guo et al., 1992), *NCDE3* (Pandey and Gautam, 2020), *NAC11* (An et al., 2018), *WRKY13* (Xiao et al., 2013), *DREB2A* (Agarwal et al., 2007), *MYB84* (Agarwal et al., 2020), *bZIP44* (Yang et al., 2019), and *KIN1* (Wang et al., 1995) play significant roles in salt and drought stresses. Analysis of the transcripts of these marker genes between EV and the soybean plants with *GmC2-148* transgenic hairy roots revealed that the soybean plants with *GmC2-148* transgenic hairy roots produced more transcripts of all of these genes under abiotic stress conditions (Figure 13). Thus the relationship between these marker genes and C2 domain family in resisting abiotic stress is worthy of further study.

## Discussion

C2 domain proteins are abundant, however, their functions are still elusive (Antal et al., 2015). In order to gain more insights, we analyzed abiotic stress responses of C2 domain genes by obtaining transcriptome data from soybean exposed to salt, drought, and ABA treatments. To clarify the functions of C2 domain genes, we isolated and identified the *GmC2-148* gene from 180 soybean C2 domain genes. The observed phenotypes and potential molecular mechanisms of *GmC2-148* demonstrate that this gene improved salt and drought tolerance in soybean.

When plants are subjected to various stresses, a large amount of active oxygen is produced. A primary mechanism of harm to plants is the imbalance of the production and removal of active oxygen in the plant body (Adamipour et al., 2020). The imbalance between systems may cause excessive accumulation of reactive oxygen species, which in turn damages proteins, membrane lipids and other cellular components, as well as causes oxidative damage to plants. Our results showed that *GmC2-148* showed strong responses to the salt, drought, and ABA treatments, as well as displayed a certain degree of response to BR. As reported, BR can enhance plant resistance to abiotic stresses through its effect on cell membranes (Marin-de la Rosa et al., 2019), and *GmC2-148* protein is located in on the cell membrane. We suspect that the response of *GmC2-148* to abiotic stresses may be involved in the BR signaling pathway. Brassinolide is a sterol plant hormone that participates in plant growth, various physiological reactions and resistance to oxidative stress development and various physiological reactions. It is also plays an essential role in plant resistance to oxidative stress. Reportedly, BR enhances the tolerance to oxidative stress by promoting the synthesis of another plant hormone, ABA, and inducing the antioxidant defense system. However, it is not clear how BR interacts with ABA to enhance C2 domain proteins in regards to plant tolerance to oxidative stress. Our research found that the gene expression levels of *GmC2-148* were simultaneously induced by BR and ABA. Therefore, we assume that C2 domain proteins may be involved in the crosstalk of various stresses and hormones in plants.

The plant root functions to absorb water and minerals provide them to stems and leaves, while storing nutrients (Zhao et al., 2020). In order to maintain and improve plant vigor in salt and drought conditions and thus increase chances of survival, roots help maintain ion balance by controlling ion migration into or out of cells through the cell membrane. A large number of studies have shown that the expression of C2 domain genes are unlike in diverse plants, for example, *CaSRC2-1* was mainly expressed in roots (Kim et al., 2008). Yang et al. (2008) found that rice *OsPBP1* expression was highest in non-pollinated pistils. We found the expression of *GmC2-148* was highest in roots (Figure 5). These results altogether suggest that C2 domain genes have different tissue specificities. We observed root length of the soybean plants with *GmC2-148* transgenic hairy roots were significantly longer than that of EV after salt and drought treatments (Figure 10), indicating that C2 domain genes respond to abiotic stresses and may enhance soybean’s ability to resist abiotic stresses. We suspect that *GmC2-148* can increase the osmotic balance of water and the balance of anions and cations in cells throughout the root under stressful circumstances, which can improve salt and drought tolerance in soybean. We also did root length experiments in *Arabidopsis* under NaCl, PEG and ABA treatments to verify the function of *GmC2-148* (Figure 11). The results showed that the *GmC2-148* transgenic plants in *Arabidopsis* improved the growth status of *Arabidopsis* compared with WT under these conditions. Shinozaki et al. have studied in detail the regulation of gene expression in *Arabidopsis* under abiotic stresses. They believe that there are at least four independent signal transduction pathways between the initial signal of dehydration stress and gene expression 2 are ABA-dependent and 2 are ABA-independent (Nakashima and Yamaguchi-Shinozaki, 2013). We speculate that C2 domain family members are ABA-dependent under abiotic stresses and gene expression. So the longer roots of *Arabidopsis* after ABA treatment compared to control WT suggested that the *GmC2-148* may act in stress tolerance through/reduction of ABA signaling or responses. The relationship between C2 domain genes and ABA will be the focus of our next experimental study.

In plants, the C2 domain proteins are reportedly involved in diverse processes, including abiotic stress, pollen fertility, and membrane targeting and leaf senescence in a Ca^2+^-dependent manner (Kang et al., 2013). Previous studies have shown that C2 domain proteins can interact with Ca^2+^ ions because of their five conserved aspartic acid (Asp) residues (Rizo and Sudhof, 1998). As report goes that Ca^2+^ plays a vital role as a second messenger in response to abiotic stresses (Zheng et al., 2014). Elevation of cytosolic Ca^2+^ is induced by salt and high osmotic pressure, and the elevated level is connected to the expression of stress-responsive genes (Zhao et al., 2010). Through our research we found that although plant C2 domain genes are not well known, a number of reports suggested their function participation in Ca^2+^ signaling (Xie et al., 2011). The SYT1, an *Arabidopsis* C2 domain protein, involved in response to freeze stress with Ca^2+^-dependent manner (Yamazaki et al., 2008). In this study, the mode of binding between C2 domain protein *GmC2-148* and Ca^2+^ needs further elaboration.

## Conclusion

In the present study, we implemented a genome-wide analysis of the C2 domain gene family in soybean. A total of 180 C2 domain genes were identified based on the conserved C2 domain, and the genes were classified into three major groups, I, II, and III. Most members of group I responded to salt, drought and ABA treatments. *GmC2-148*, encoding a cell membrane-localized protein, is a positive regulator of plant tolerance to salt and drought stresses by activating abiotic stress-related genes. However, further study is needed to determine the specific functions of the C2 domain genes. To sum up, the present study investigates the importance of C2 domain genes in abiotic stress-tolerance in soybean.

## Data Availability Statement

The raw data supporting the conclusions of this article will be made available by the authors, without undue reservation.

## Author Contributions

Z-SX coordinated the project, conceived and designed the experiments, and edited the manuscript. YS performed the experiments and wrote the first draft. J-YZ revised the manuscript. Y-TL and P-GZ contributed to the data analysis and managed reagents. Z-WF contributed with the valuable discussions. JG put forward valuable opinions when revising the manuscript. All authors reviewed and approved the final manuscript.

## Conflict of Interest

The authors declare that the research was conducted in the absence of any commercial or financial relationships that could be construed as a potential conflict of interest.

## Footnotes

1 https://phytozome.jgi.doe.gov/pz/portal.html

2 https://www.ebi.ac.uk/Tools/hmmer/

3 http://www.ncbi.nlm.nih.gov/cdd

4 http://pfam.xfam.org/

5 http://smart.emblheidelberg.de/

6 https://www.expasy.org/

7 http://www.genome.jp/tools-bin/clustalw

8 https://sourceforge.net/projects/mcscanx/

9 http://gsds.cbi.pku.edu.cn/

10 http://meme-suite.org/tools/meme

11 http://www.tbtools.com/

12 https://soybase.org/soyseq/

13 https://www.dna.affrc.go.jp/PLACE/action=newplace

14 http://www.blast2go.com

## Supplementary Material

Supplementary Figure 1 Chromosomal location of C2 domain genes. Blue bars represent the chromosomes with their chromosome number above the bar. The names of C2 domain genes are labeled at both sides of the chromosomes. Scale bars on the left show chromosome lengths (Mb).

Supplementary Figure 2 Putative *cis*-elements in a 1.5-kb 5′-flanking region upstream from the start codon. Various *cis*-elements are indicated by colored symbols and placed in positions relative to the promoter.

Supplementary Figure 3 GO enrichment annotation map.

Supplementary Figure 4 Annotation diagram of *GmC2-148* C2 domain.

Supplementary Figure 5 Annotated diagram of *GmC2-148* transmembrane protein.

Supplementary Table 1 Physicochemical properties related description of C2 domain genes in soybean.

Supplementary Table 2 Primers and sequences used in this study.

Supplementary Table 3 Data about the response of C2 domain genes in salt treated transcriptome.

Supplementary Table 4 Data on the response of C2 domain genes in drought treated transcriptome.

Supplementary Table 5 Data on the response of C2 domain genes in ABA treated transcriptome.

Supplementary Table 6 Data of tissue-specific expression levels of C2 domain genes.

## Abbreviations

ABA: abscisic acid
RT-qPCR: real-time quantitative polymerase chain reaction
GFP: green fluorescent protein
MDA: malondialdehyde
Pro: proline
PEG: polyethylene glycol
BR: brassinolide
CDS: coding sequence length
HMM: Hidden Markov model
NJ: neighbor-joining
EV: empty vector
NBT: Nitrogen Blue Tetrazole
WT: wild type.
