# Supplementary figures and images for: Genome-Wide Analysis of the C2 Domain Family in Soybean and Identification of a Putative Abiotic Stress Response Gene GmC2-148
Source: Front Plant Sci. 2021 Feb 16;12:620544. doi: 10.3389/fpls.2021.620544 (PMC7939022; doi:10.3389/fpls.2021.620544)

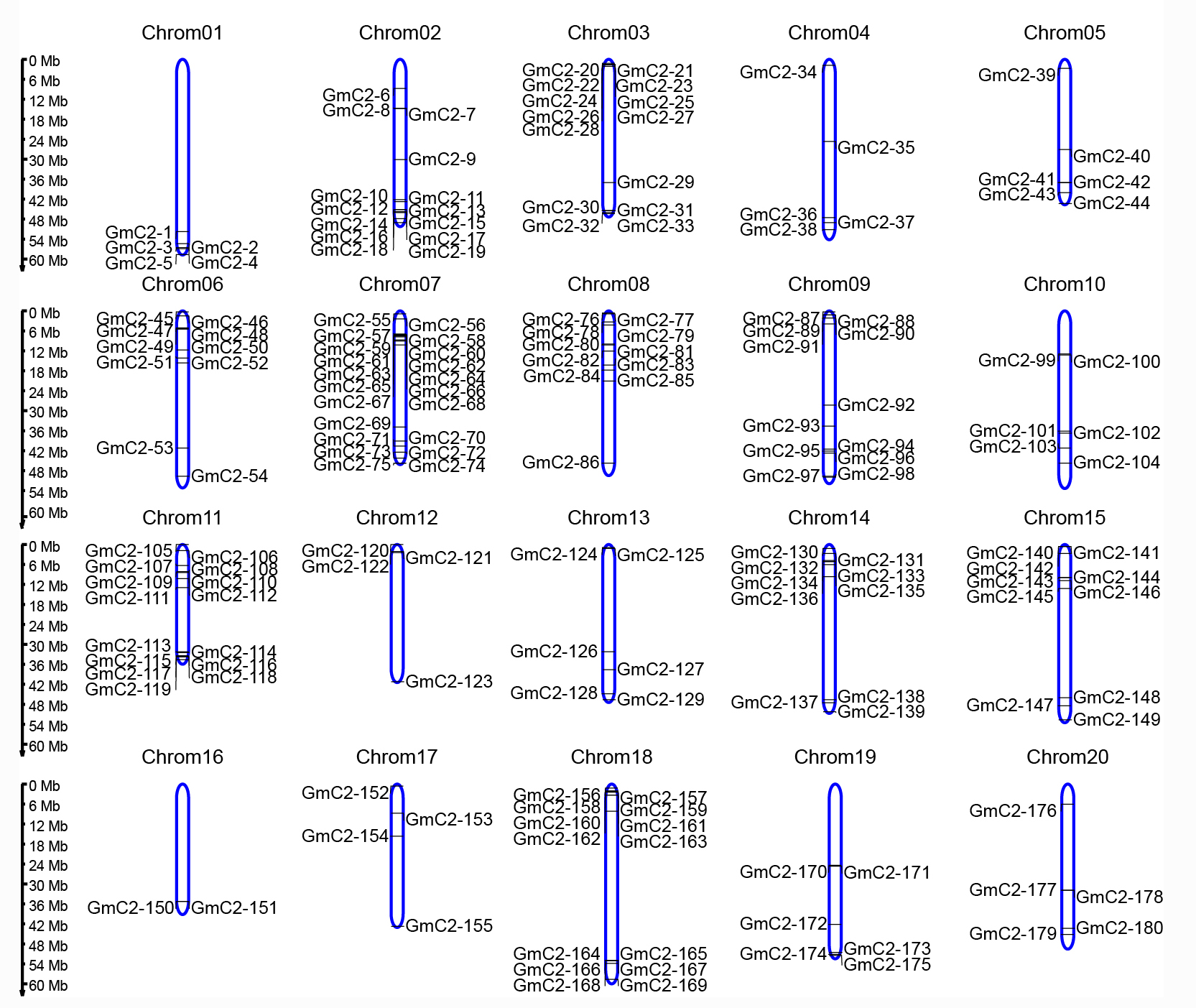

Supplement: Supplementary Figure 1 — Chromosomal location of C2 domain genes. Blue bars represent the chromosomes with their chromosome number above the bar. The names of C2 domain genes are labeled at both sides of the chromosomes. Scale bars on the left show chromosome lengths (Mb). [file Image_1.JPEG]

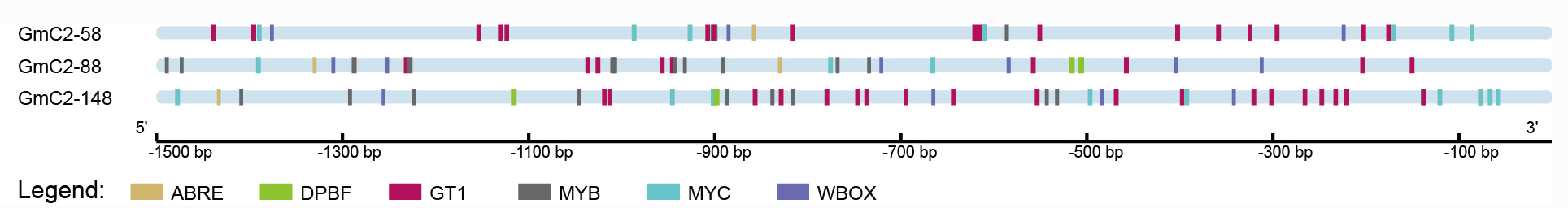

Supplement: Supplementary Figure 2 — Putative cis-elements in a 1.5-kb 5′-flanking region upstream from the start codon. Various cis-elements are indicated by colored symbols and placed in positions relative to the promoter. [file Image_2.JPEG]

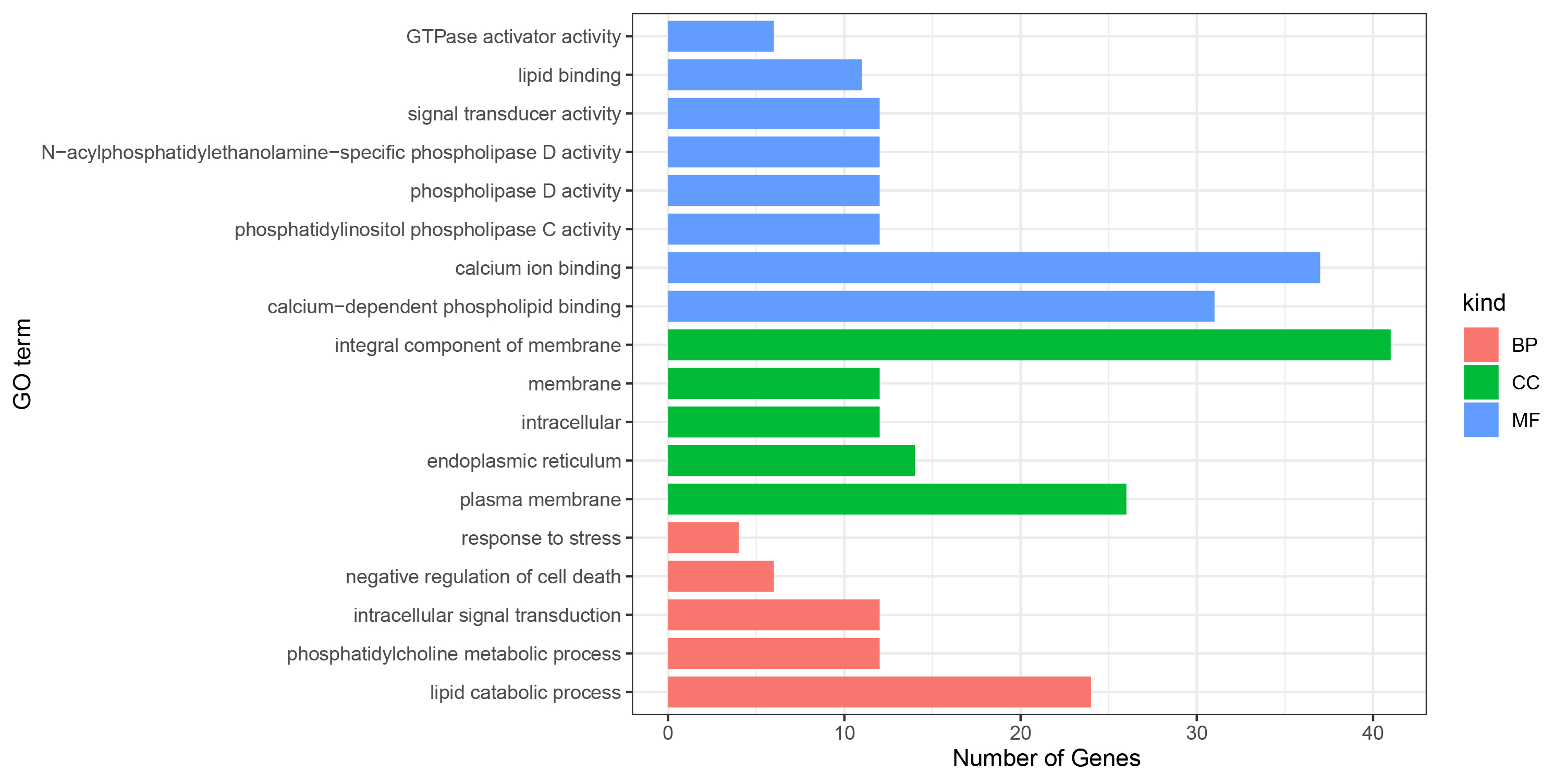

Supplement: Supplementary Figure 3 — GO enrichment annotation map. [file Image_3.JPEG]

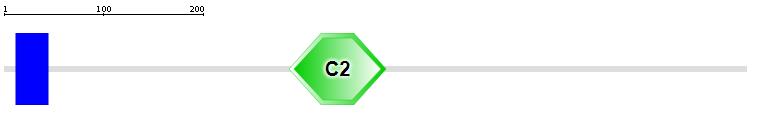

Supplement: Supplementary Figure 4 — Annotation diagram of GmC2-148 C2 domain. [file Image_4.JPEG]

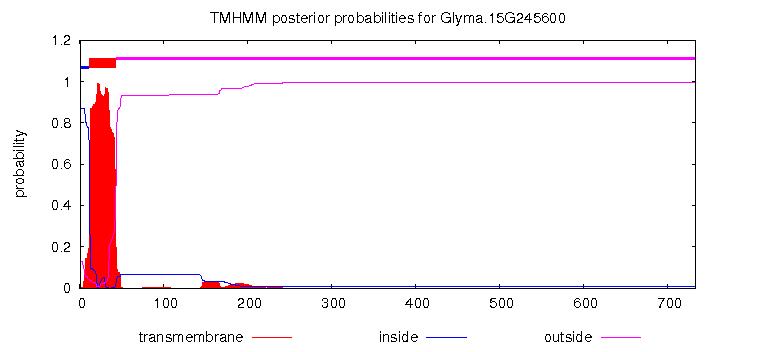

Supplement: Supplementary Figure 5 — Annotated diagram of GmC2-148 transmembrane protein. [file Image_5.JPEG]
