# Supplementary material for: Genome-Wide Analysis of the C2 Domain Family in Soybean and Identification of a Putative Abiotic Stress Response Gene GmC2-148
Source: Front Plant Sci. 2021 Feb 16;12:620544. doi: 10.3389/fpls.2021.620544 (PMC7939022; doi:10.3389/fpls.2021.620544)
Supplement: Supplementary Table 1 — Physicochemical properties related description of C2 domain genes in soybean. [file Table_1.DOCX]

| Gene | Sequences |
| --- | --- |
| Soybean actin F | ACATTGTTCTTAGTGGTGGCT |
| Soybean actin R | CTGTTGGAAGGTGCTGAG |
| GmC2-148 F | GGCATGCATTTCGCGTGTTA |
| GmC2-148 R | AGCACCATCTGAAACCCGAA |
| RTGmC2-58 F | TGCTGGATCCAAGCCACAAT |
| RTGmC2-58 R | ACCCGCTTCAAACACTGGAT |
| RTGmC2-88 F | TTGAAGATAATGGAAAGGGA |
| RTGmC2-88 R | TTCCTGGTCACGTGTAGTAG |
| RTGmC2-148 F | AGGAAAGGGAAGAGTCGG |
| RTGmC2-148 R | GCCTGATTTACAAGAAAGAACGT |
| GmC2-148 -3301 F | GGACTCTTGACCATG GGCATGCATTTCGCGTGTTA |
| GmC2-148 -3301 R | ATTCGAGCTGGTCACC TCATTGAGACATCTGCCC |
| GmC2-148 -GFP F | TATCTCTAGAGGATCC GGCATGCATTTCGCGTGTTA |
| GmC2-148 -GFPR | TGCTCACCATGGATCCATGCATTTCGCGTGTTA |
| GmCOR47 F | TTTTGTTGGTTGGAGATGCATG |
| GmCOR47 R | AAGAAACACCAAAGAAACCCAC |
| GmNCED3 F | GAAGAGCATCTTGTCGGAGATA |
| GmNCED3 R | CTAAGTACCCGAACTTCGTCTT |
| GmNAC11F | TCCCTCTTGTTCTATGCT |
| GmNAC11R | GTTTCCCATTATTTGCCTA |
| GmWRKY13F | AGGACACGGATACAGCAA |
| GmWRKY13R | CATCTTCAGGGTAGGCAC |
| GmDREB2A F | AGCGAAAGCAGCAGCACC |
| GmDREB2A R | GTTAAGGCGAGCGGAAGG |
| GmMYB84 F | GGGGAAACAGGTGGTCAA |
| GmMYB84 R | TCTAGGCATCCAGAAACG |
| GmbZIP44 F | TCGGATGCGAAAGCGTAA |
| GmbZIP44 R | TGCGTGGTGATGTCTATGGTG |
| GmKIN1F | CCACTGCTCTTTTCTCGAGATA |
| GmKIN1R | TTTCTTCGTACACACTAGCTCC |
